# Modern Treatment of Valvulopathies in Patients with Congenital Hemophilia

**DOI:** 10.3390/life14030354

**Published:** 2024-03-07

**Authors:** Minerva Codruta Badescu, Oana Viola Badulescu, Liliana Gheorghe, Lăcrămioara Ionela Butnariu, Anca Ouatu, Diana Popescu, Oana Nicoleta Buliga-Finiș, Eusebiu Vlad Gorduza, Manuela Ciocoiu, Ciprian Rezus

**Affiliations:** 1 Department of Internal Medicine, “Grigore T. Popa” University of Medicine and Pharmacy, 700115 Iasi, Romania; minerva.badescu@umfiasi.ro (M.C.B.); anca.ouatu@umfiasi.ro (A.O.); popescu.diana@umfiasi.ro (D.P.); oana-nicoleta.buliga-finis@umfiasi.ro (O.N.B.-F.); ciprian.rezus@umfiasi.ro (C.R.); 2 III Internal Medicine Clinic, “St. Spiridon” County Emergency Clinical Hospital, 700111 Iasi, Romania; 3 Department of Pathophysiology, “Grigore T. Popa” University of Medicine and Pharmacy, 700115 Iasi, Romania; oana.badulescu@umfiasi.ro (O.V.B.); manuela.ciocoiu@umfiasi.ro (M.C.); 4 Hematology Clinic, “St. Spiridon” County Emergency Clinical Hospital, 700111 Iasi, Romania; 5 Department of Radiology, “Grigore T. Popa” University of Medicine and Pharmacy, 700115 Iasi, Romania; 6 Radiology Clinic, “St. Spiridon” County Emergency Clinical Hospital, 700111 Iasi, Romania; 7 Department of Mother and Child Medicine, “Grigore T. Popa” University of Medicine and Pharmacy, 700115 Iasi, Romania; eusebiu.gorduza@umfiasi.ro

**Keywords:** hemophilia, cardiac surgery, valvular disease, valvular prosthesis, valvular repair, anticoagulants, antiplatelet agents

## Abstract

Hemophiliacs can develop cardiovascular diseases, including valvulopathies of various etiologies and severities. Some require surgical treatment. Performing cardiac surgery in hemophiliacs is a challenge because they maintain an increased risk of bleeding throughout their lives. Our review shows that with a multidisciplinary team and careful planning, cardiac surgery can be safely performed in these patients. Valve repair and bioprosthetic valves should be preferred over mechanical valves to avoid life-long anticoagulation. In patients who cannot receive a bioprosthetic valve, the use of the On-X mechanical valve might be considered because it requires less intensive anticoagulation after 3 months of treatment. Antithrombotic treatment is feasible in hemophiliacs only if the coagulation factor level is kept constantly above a specific trough limit. Our review is valuable because, for the first time, the available data on the modern surgical treatment of valvular disease in hemophiliacs have been synthesized and systematized.

## 1. Introduction

Due to the increase in life expectancy and long-term action of cardiovascular risk factors, atherosclerosis and its complications have become more and more common in hemophiliacs [1]. There is already successful experience in performing primary or elective percutaneous coronary intervention (PCI) with stent implantation and open-chest surgery for coronary artery bypass grafting (CABG) in this high-bleeding-risk population [2,3]. Valvular damage, of various etiologies and varying degrees of severity, is also part of the spectrum of cardiovascular diseases in hemophiliacs [4,5,6,7]. Severe forms that necessitate surgical intervention have the most difficult therapeutic management.

Major cardiac surgery generally requires cardiopulmonary bypass (CPB). This procedure represents a significant hemostatic challenge in patients with hemophilia, mainly due to extracorporeal circulation and total heparinization, which considerably alter normal hemostasis. During CPB, there are both dilutional and consumptive losses of platelets and coagulation factors [8]. Clinically significant reductions in platelets, fibrinogen, FII, FV, FX, FXIII, and antithrombin III levels of 35% to 49% were reported after CPB [9]. In non-hemophilic patients, the FVIII level is maintained due to the release of factors stored in endothelial cells. In hemophiliacs, however, despite the preoperative correction of the FVIII level, a significant reduction associated with CPB was identified (from 83% to 43%), in a pattern that mimics the behavior of other coagulation factors [8]. 

As it is very difficult to maintain a hemostatic balance in conditions of anticoagulation, blood loss, hemodilution, and replacement therapy of coagulation factors, the challenges of major cardiac surgery in hemophiliacs are emphasized by all the literature published so far. Because hemophilia is a rare disease and open-heart surgery is even rarer, there are no evidence-based guidelines from randomized controlled trials or large-scale observational studies to be implemented in practice. Retrospective analyses of published articles and institutional experience struggle to provide a reliable framework for cardiac surgery in this category of high-bleeding-risk patients [10,11].

Our review focused only on the adult population and had several objectives. Because it is easier to prevent than to treat, the first objective was the identification and characterization of the valvular substrate that required these complex and difficult surgical interventions. Given that valvular lesions may require repair or total valve replacement, another objective was to identify the types of devices and implantation methods used. We restricted our search to English literature published since 2000, to reflect the modern approach to valvular diseases, taking into account the continuous progress in the field, such as new devices, robotic cardiovascular surgery, and transcatheter valvular interventions. Considering that cardiac surgeries in hemophiliacs require the administration of large amounts of coagulation factors, we investigated whether these interventions lead to the occurrence of inhibitors. This is an important aspect because the presence of inhibitors changes the subsequent therapeutic management of hemophilia. Another point of interest is long-term antithrombotic therapy, which we also considered challenging in patients with congenital anomalies of hemostasis. 

## 2. Etiological Spectrum of Valvular Lesions

In adult hemophiliacs, only interventions for left heart valve diseases were published. In some patients, the pathology was complex, involving both the aortic valve and the ascending aorta. The available data came from case reports and two very short series of patients. Surprisingly, there was a wide etiological spectrum, although many authors reported only the implantation of the prosthesis or the valve repair and not the pre-existing valvular lesions. The youngest patients, aged between 23 and 27, underwent surgery for rheumatic valvular disease [4,12,13]. Three cases of infective endocarditis (IE) were reported, two affecting the aortic valve (AV) [6,14] and one the mitral valve (MV) [15]. Significant mitral regurgitation (MR) was reported in six patients. One of them had MV prolapse and moderate-to-severe MR due to rupture of a tendinae [5]. In two patients, MV flail was identified [16,17]. Aortic valve replacement (AVR) for stenosis was performed in four patients [18,19,20,21], one of them with a bicuspid valve. Aortic regurgitation (AR) requiring valvular replacement was mostly associated with diseases of the aortic root and ascending aorta. A Bentall operation or modified Bentall operation was performed in three patients with dilated aortic root and/or ascending aorta [22,23,24] and in one patient with acute type-A aortic dissection [25].

The etiological spectrum of valve disease in hemophiliacs parallels that of individuals in the general population. Valvular insufficiency was more prevalent than stenosis, usually caused by structural degeneration. Although IE seems more common in the elderly [26], hemophiliacs with IE who required valve replacement were younger than 60 years old. 

## 3. Prosthetic Cardiac Devices

Patients with hemophilia required a wide range of interventions, namely MV repair, MV replacement, AV replacement, and complex surgery such as the Bentall procedure (Table 1, Table 2, Table 3 and Table 4).

Few mitral prostheses were implanted. Except for a 54-year-old patient who underwent a redo mitral valve replacement (MVR) and AVR with biological prostheses [27], the other patients received mechanical prostheses [4,13,28]. One patient was a 53-year-old hemophiliac with mild disease, who required both MV replacement and triple CABG [28], and two patients were young hemophiliacs, 25 and 27 years old, respectively, with rheumatic valvular disease [4,13]. Because mechanical prosthesis requires life-long anticoagulant treatment, we emphasize that all patients received a vitamin K antagonist (VKA), without hemorrhagic complications in the short-term follow-up.

Most interventions on MV were repairs. In two cases, MV repair was performed concurrently with CABG [5,29], and in one case, with the resection of a left ventricular aneurysm [30]. Artificial chordae were necessary for three patients [5,16,17]. Insertion of an annuloplasty system—ring or band—was performed in five patients [5,15,16,17,29]. In general, recovery after MV repair interventions was uneventful. One patient had minor surgical bleeding managed conservatively [7], and another patient had a right pleural effusion requiring repeated thoracentesis to relieve symptoms [17].

Among all valve surgeries, aortic valve replacement was the most frequent. Few hemophiliacs were deemed to receive mechanical aortic prostheses [13,27,31]. While two of them had an uneventful recovery, the 25-year-old hemophiliac who underwent mechanical replacement of both mitral and aortic valves developed cardiac tamponade requiring urgent treatment. All patients with mechanical prostheses received a VKA. However, a clear preference for the use of biological valves was observed. Moreover, concomitant CABG and aortic valve replacement were quite common. The Bentall procedure—classic or modified—was necessary in a few cases, and composite grafts with either biological or mechanical aortic valves were used. 

There are pros and cons for each type of valve prosthesis [32]. Mechanical prostheses last the rest of the person’s life. Therefore, implantation of a mechanical valve requires only a single surgical procedure and there is no need to repeat the cardiac surgery later in the patient’s life. The disadvantage is the mandatory permanent anticoagulant treatment. Furthermore, because only VKAs are allowed, there is a struggle to maintain the INR in the therapeutic range, as supratherapeutic values increase the risk of bleeding and subtherapeutic values increase the risk of prosthesis thrombosis.

Biological prostheses have superior hemodynamic properties compared to mechanical prostheses and are free from the burden of long-term anticoagulant treatment because they are less thrombogenic. Depending on the method of implantation—surgical or transcatheter—and on the implanted valve—mitral or aortic—antithrombotic therapy with VKA or aspirin is short-term, of 3 months [32]. The disadvantage is the limited durability due to the degeneration of the valves and, consequently, the need for reintervention. In the mitral position, bioprosthetic valves are expected to degenerate faster than in the aortic position. The lifespan of a surgically inserted bioprosthesis is 10–20 years [33]. Although little is known about the long-term durability of transcatheter-implanted prostheses, recent data show that the hemodynamic function of transcatheter heart valves remains stable up to more than 10 years post-TAVR [34]. The possibility of a valve-in-valve procedure using a transcatheter approach pushes the limits further. A new suggested algorithm for the first-line treatment of bioprosthetic valve dysfunction proposes that structural valve deterioration should be percutaneously treated [33]. It is even speculated that future strategies of repeated TAVR procedures could allow the avoidance of the use of mechanical prostheses or surgical valve replacement [33].

**Table 1:** Mitral valve repair in patients with hemophilia A.

Author, Year	Patient Characteristics	Indication for Valvular Surgery	Procedure	Comorbidities	Outcome	Antithrombotic Treatment after Surgery
Xu et al., 2019 [5]	54 years HA severe I—NR	MV prolapse (posterior leaflet, mainly P2) + moderate-to-severe MR due to rupture of tendinae	MV repair: triangular resection of the posterior leaflet, creation of an artificial chordae (Gortex CV-4), insertion of an annuloplasty ring (Sorin, no. 28) + CABG	60% stenosis in the proximal LAD; LVEF 70%; bilateral knee replacement	uneventful recovery; discharged home on POD 8; one-year follow-up: no bleeding or thrombosis complications; no MR on TTE; I—NR	life-long aspirin (due to CABG)
Odonkor et al., 2017 [8]	59 years HA mild I—no	symptomatic MR	minimally invasive MV repair	chronic hepatitis C	uneventful recovery; discharged home on POD 9; I—NR	aspirin
Bhave et al., 2015 [7]	59 years HA moderate I—no	NR	MV repair	NR	wound ooze and small retrosternal hematoma on POD 12 managed conservatively; aspirin withheld temporarily; I—bleeding phenotype typical of inhibitor development absent during the follow-up period	aspirin
Tran et al., 2015 [17]	60 years HA moderate I—yes (high-titer inhibitor: 12.4 BU)	MR + flail of the posterior leaflet	mitral valve repair: Gore-Tex neochord to the P3 segment; 28 mm Simulus ring around the mitral annulus; suture ligation of the left atrial appendage; pulmonary vein isolation using cryoablation; endoscopic robotic approach (da Vinci telemanipulation system)	HF with preserved LVEF	pleural effusion requiring ultrasound-guided thoracentesis (POD 3, POD 16, POD 27); I: high-titer inhibitor (6.2 BU) at 6-month follow-up	none
Zatorska et al., 2012 [15]	30 years HA mild I—no	IE of the MV with *Staphyloccocus aureus* MSSA; perforation of the posterior MV leaflet; prolapse of the anterior MV leaflet; considerable MR (acute)	triangular resection of anterior leaflet; quadrangular resection of the posterior leaflet; mitral valve annuloplasty with ring implantation (Edwards Phisio, 28 mm)	arthroscopic surgery for post-traumatic chondral and ligamentous damage of the left knee; bacterial pneumonia; left HF	no bleeding complications; the valve functioned very well at 1-year follow-up; I—NR	acenocoumarol, target INR 2–3, for 4 weeks (starting from POD 7), then aspirin for 2 months
Tang et al., 2009 [30]	72 years HA mild I—no	NR	LV aneurysm resection + MV repair	hypertension; congestive HF; LVEF = 30–35%	I—no (3-month follow-up)	aspirin
Stine et al., 2006 [29]	64 years HA mild I—NR	significant MR	MV repair (annuloplasty ring) + CABG	one stenotic coronary artery	no bleeding; no thrombosis; I—NR	aspirin and coumadin 7.5 mg each day for 30 days

HA = hemophilia A; I = inhibitors; IE = infective endocarditis; POD = postoperative day; HF = heart failure; MV = mitral valve; MR = mitral regurgitation; CABG = coronary artery bypass grafting; LAD = left anterior descending artery; LVEF = left ventricular ejection fraction; TTE = transthoracic echocardiography; MSSA = methicillin-sensitive *Staphylococcus aureus*; INR = international normalized ratio; LV = left ventricle; NR = not reported.

**Table 2:** Mitral and aortic valve replacement in patients with hemophilia A.

Author, Year	Patient Characteristics	Indication for Valvular Surgery	Procedure	Comorbidities	Outcome	Antithrombotic Treatment after Surgery
Surgically implanted biological prostheses						
Cusano et al., 2022 [6]	54 years HA severe I—NR	group B *Streptococcus* IE + aortic root abscess + AR	AVR + bovine patch reconstruction + aortic root repair	hepatitis C (treated); Child–Pugh B cirrhosis; HIV on antiretroviral therapy (undetectable viral load); GI bleeding treated with FEIBA and tranexamic acid, complicated by portal vein thrombosis	no excessive intraoperative bleeding; chest reopening for bleeding from the sternal bone and muscle (OD); no further bleeding until POD 14; I—NR	none
Serban et al., 2022 [35]	64 years HA severe I—no	large AR	AVR	HF	discharged POD 10 immediate and long-term outcomes were good, without complications; I—NR	3 months LMWH, then 3 months aspirin
Chamos et al., 2017 [14]	57 years HA severe I—no	severe AR secondary to IE with *Staphylococcus epidermidis* antibiotics iv for 6 weeks	AVR with 23 mm Perimount Magna Ease bioprosthetic valve through an anterior right thoracotomy	hemophilic arthropathy; HIV; HCV; hemodialysis thrice weekly for AKI	no bleeding complications associated with the procedure; renal function recovered completely; discharged home on POD 10; I—NR	none; allergy to aspirin and penicillin
Bhave et al., 2015 [7]	NR HA mild I—no	NR	AVR	NR	no complications; I—bleeding phenotype typical of inhibitor development absent during the follow-up period	NR
	NR HA severe I—no	NR	AVR + CABG	NR	no complications; discharged to a rehabilitation facility due to significant de-conditioning; I—bleeding phenotype typical of inhibitor development absent during the follow-up period	NR
Damodar et al., 2014 [12]	23 years HA severe I—yes (low-titer inhibitor 2.8 BU)	severe rheumatic AR	AVR	HF	uneventful recovery; discharged on POD 15; normal AV function, without any complications at 1-year follow-up; I—reevaluation of the titer was not considered necessary	heparin iv infusion, 48 h; LMWH for 10 days; discharged without anticoagulant
Fitzsimons et al., 2013 [36]	53 years HA mild I—no	redo aortic valve replacement	AVR	aortic valve replacement (1997); antiphospholipid antibody syndrome; hepatitis C; hematuria; complex migraine headaches; grand mal and petit mal seizures; IgA deficiency; anaphylactic reaction after a blood transfusion; chronic pain (requiring methadone); hemolytic anemia; idiopathic thrombocytopenia from interferon treatment; splenectomy	cardiac tamponade 6 h postoperatively; chest left open for 4 days to ensure hemostasis; discharged to rehabilitation on POD 27; 1-year follow-up: good exercise tolerance, no cardiac symptoms, minimal trouble with swallowing; I—NR	none
Quader et al., 2013 [37]	63 years HA mild I—NR	moderate AR	LVAD (HeartMate II) + AVR 23 mm Magna valve + CABG heart transplant (after 156 days)	ischemic cardiomyopathy; severe MR; moderate TR; advanced HF (severe biventricular failure); elevated pulmonary artery pressures; cardiogenic shock; multivessel coronary artery disease; motor vehicle accident 20 years previously: repair of the left temporal artery associated with excessive bleeding	HF symptoms improved; purse-string skin stitches to contain oozing from insertion site of chest tubes after their removal; multiple episodes of GI bleeding; possible pump thrombosis; transient ischemic attacks; discharged on POD 22 after heart transplant; good health at 4-month follow-up; I—NR	warfarin from POD 2; changed to heparin due to recurrent bleeding and thrombotic events
Lison et al., 2011 [19]	67 years HA moderate I—NR	AS and mild AR	AVR + CABG	two-vessel disease with 60% left main stem occlusion	uneventful recovery; discharged on POD 7; I—NR	none
Tang et al., 2009 [30]	60 years HA mild I—no	NR	AVR	hypertension, LVEF = 35–40%	postoperative duodenal ulcer 13 days after discharge, requiring readmission; I—no (3-month follow-up)	LMWH 6 weeks postoperatively (stopped because of GI bleeding)
	68 years HA moderate I—no	NR	AVR + CABG	atrial fibrillation; hypertension; hypercholesterolemia; LVEF = 40%	I—no (3-month follow-up)	LMWH 3 months
	61 years HA mild I—no	NR	AVR + CABG	hypertension; hypercholesterolemia; LVEF = 55%	atrial flutter, sinus rhythm 1 month after surgery; I—no (3-month follow-up)	LMWH 1 month
Gasparovic et al., 2007 [18]	47 years HA severe I—no	critical aortic stenosis	AVR	clinical stigmata of severe hemophilia	uneventful recovery	none
Mackinlay et al., 2000 [27]	54 years HA moderate I—no	second valvular replacement	AVR + MVR	AVR + MVR (bioprostheses) at the age of 49	surgical bleeding due to severe uncontrolled hypertension, requiring reintervention; I—no	none
Transcatheter aortic bioprostheses						
Merron et al., 2015 [20]	84 years HA mild I—no	severe AS with recurrent exacerbations of acute HF	TAVR (transfemoral)	transient ischemic event; stented stenosis of left ICA; quadruple CABG; large spontaneous gluteal bleed while on aspirin without FVIII prophylaxis; congestive HF; left ventricular dysfunction; type 2 diabetes mellitus; essential hypertension; hiatus hernia repair; cholecystectomy; bilateral total hip replacement and subsequent revision	no excessive bleeding; discharged on POD 21; I—NR	aspirin
Surgically implanted mechanical prostheses						
Mannucci et al., 2010 [31]	45 years HA mild I—NR	NR	AVR	NR	no postoperative bleeding complications; no increase in hemorrhagic tendency; I—NR	life-long coumarin (target INR 2.5–3.5)
De Bels et al., 2004 [28]	53 years HA mild I—NR	grade III MR	MVR (Carbomedics mechanical prosthesis) + CABG	arterial hypertension; stable angina pectoris; two-vessel disease (occlusion RCx, triple stenosis LAD)	discharged on POD 9; I—NR; no bleeding complication at 6- week follow-up	coumarin
Ghosh et al., 2003, 2004 [4,38]	27 years undiagnosed HA mild	severe rheumatic MV stenosis previously treated by balloon valvuloplasty	MVR	large hematoma at the puncture site at the time of balloon mitral valvuloplasty; hematomas at the site of intramuscular benzathine penicillin injections	excessive bleeding and persistent hypotension during surgery; after surgery: hemoperitoneum; pericardial hematoma without tamponade; right-sided hemothorax; shock; ventilatory support; I—no; hematoma in right upper and mid thorax; I—yes (2.4 BU); no bleeding at 1-month follow-up after the start of VKA (INR 1.3–1.6) + I—yes (<2 BU); patient is well at 2.5-year follow-up, on VKA (INR 1.3–1.8)	discharged without anticoagulant; warfarin 1 mg/day (target INR 1.5–2) started 3 months after the operation; last report: warfarin 2.5 mg/day
Mackinlay et al., 2000 [27]	58 years HA mild I—no	NR	AVR (Medtronic Hall valve)	NR	no major bleeding complications related to warfarin therapy; I—no	warfarin (target INR 2–2.5)

HA = hemophilia A; I = inhibitors; IE = infective endocarditis; AV = aortic valve; AS = aortic stenosis; AR = aortic regurgitation; AVR = aortic valve replacement; MV = mitral valve; MR = mitral regurgitation; MVR = mitral valve replacement; TAVR = transcatheter aortic valve replacement; LVEF = left ventricular ejection fraction; HF = heart failure; LAD = left anterior descending artery; RCx = circumflex artery; CABG = coronary artery bypass grafting; LVAD = left ventricular assist device; ICA = internal carotid artery; TR = tricuspid regurgitation; IgA = immunoglobulin A; HIV = human immunodeficiency virus; HCV = hepatitis C virus; AKI = acute kidney injury; GI = gastrointestinal; FEIBA = factor eight inhibitor bypass activity; LMWH = low-molecular-weight heparin; INR = international normalized ratio; VKA = vitamin K antagonist; OD = operation day; POD = postoperative day; NR = not reported.

**Table 3:** Cardiac surgery involving the aortic valve and the ascending aorta in patients with hemophilia A.

Author, Year	Patient Characteristics	Indication for Valvular Surgery	Procedure	Comorbidities	Outcome	Antithrombotic Treatment after Surgery
Biological aortic prostheses						
Yildirim et al., 2016 [22]	43 years HA severe I—no	grade 3/4 AR, dilated aortic root and ascending aorta	Bentall operation; biologic composite graft (Sorin Mitraflow Valsalva conduit, no. 23)	Marfan syndrome; hemarthroses of the ankles, wrists, and knees; left knee fixation; operated hepatic hydatid cyst; LVEF 60%	uneventful recovery; discharged on POD 7; I—NR	none
Bhave et al., 2015 [7]	NR HA mild I—no	NR	aortic root replacement (Valsava graft 30 mm + Carpentier Edwards Perimount Magna AV)	NR	no complications; I—bleeding phenotype typical of inhibitor development absent during the follow-up period	NR
Diplaris et al., 2012 [25]	54 years HA severe I—no	acute type A aortic dissection + bicuspid AV	Bentall operation: composite graft with a biologic valve (Biovalsalva 23)	hepatitis C infection; knee arthroplasty	re-exploration for bleeding on POD 1; sternal wound bleeding on POD 6 managed conservatively; discharged on POD 11; good condition at 3-month follow-up; I—NR	no postoperative prophylactic anticoagulation
Mechanical aortic prostheses						
Bhave et al., 2015 [7]	44 years HA mild I—no	NR	aortic root replacement (St Jude valved conduit 25 mm)	NR	frank hematuria 5 weeks postoperatively at INR 2.9; patient received TXA; INR target lowered at 2–2.5; I—bleeding phenotype typical of inhibitor development absent during the follow-up period	warfarin (target INR 2–3)
Kaminishi et al., 2003 [23]	53 years HA mild I—no	severe AR + dilation of ascending aorta	modified Bentall operation: 28 mm vascular graft (Hemashield Gold) and a 25 mm mechanical valve (Carbomedics)	shoulder trauma in a motor vehicle accident; congestive HF	no excessive bleeding; neurologically intact and cardiovascularly stable upon discharge on POD 23; I—NR	warfarin from POD 4

HA = hemophilia A; I = inhibitors; AV = aortic valve; AR = aortic regurgitation; LVEF = left ventricular ejection fraction; HF = heart failure; POD = postoperative day; TXA = tranexamic acid; INR = international normalized ratio; NR = not reported.

**Table 4:** Cardiac surgery involving heart valves in patients with hemophilia B.

Author, Year	Patient Characteristics	Indication for Valvular Surgery	Procedure	Comorbidities	Outcome	Antithrombotic Treatment
Mitral valve repair						
Miller et al., 2020 [16]	38 years HB mild	severe symptomatic MR with posteriorly directed jet related to an anterior mitral leaflet flail (Carpentier type II MV dysfunction)	robotic MV repair; five neochordae to segments A1 and A2 of the anterior mitral leaflet; 33 mm ATS annuloplasty band (Medtronic Inc) to the posterior annulus	severe LA and LV dilatation; mild AR; bicuspid AV; LVEF 55%	uneventful recovery; discharged home POD 7	none at discharge
Surgically implanted biological prostheses						
Shalabi et al., 2020 [39]	74 years HB mild	NR	AVR + CABG	risk factors for ischemic heart disease; LVEF 55%; hepatitis C	GI bleeding requiring rehospitalization; I—no	antiplatelet at discharge
Krakow et al., 2008 [21]	61 years HB mild I—no	critical aortic stenosis; bicuspid aortic valve	AVR	NR	no bleeding complications; discharged on POD 7; I—no	heparin 5000 IU sc/12 h in POD 1–7; aspirin from POD 5
Surgically implanted mechanical prostheses						
Thankachen et al., 2007 [13]	25 years HB mild	rheumatic mitral and aortic valvular disease	MVR with 2 M Starr Edwards valve (Model 6120, Edwards Lifesciences) + AVR with 22 Medtronic valve	intracerebral bleed with complete neurological recovery	cardiac tamponade (POD 4) treated by subxiphoid pericardiocentesis; acute renal failure treated conservatively (no dialysis required); discharged on POD 17; the patient was doing well at 9- month follow-up.	heparin 10 IU/Kg/h followed in POD 3–11 by dalteparin 2500 IU sc/12 h; none at discharge; acenocoumarol (INR 1.5–2.0) once the renal failure resolved completely
Surgery involving the aortic valve and the ascending aorta						
Bohn et al., 2022 [24]	60 years HB mild	degenerative aneurysms of aortic root and ascending aorta	Bentall operation type of graft—NR	uncomplicated dental extraction	uneventful recovery; I—no at 6-week follow-up	enoxaparin 40 mg/day sc from POD 1 throughout intensified FIX prophylaxis

HB = hemophilia B; I = inhibitors; IE = infective endocarditis; POD = postoperative day; MV = mitral valve; MR = mitral regurgitation; MVR = mitral valve replacement; LA = left atrium; LV = left ventricle; AV = aortic valve; AR = aortic regurgitation; AVR = aortic valve replacement; LVEF = left ventricular ejection fraction; CABG = coronary artery bypass grafting; GI = gastrointestinal; NR = not reported.

Several factors are important when choosing the type of valve prosthesis. One is the patient’s age. Younger patients benefit from mechanical prostheses while the elderly benefit from biological prostheses. There is slight variability in the age limit depending on the valve that will be replaced and between the recommendations of the European Society of Cardiology (ESC) and the American College of Cardiology/American Heart Association guidelines (ACC/AHA) [32,40]. In patients requiring mitral valve replacement aged 65–70 years (ESC) and in patients requiring aortic valve replacement aged 60–65 years (ESC)/50–65 years (ACC/AHA), both types of valves are acceptable, and valve choice requires careful consideration of factors other than age [32,40]. It should be emphasized that there is a consistent trend towards the use of biological valves in patients at the younger end of this range [33]. The patient’s life expectancy is also important because, unlike absolute age, it integrates the effect of comorbidities. A bioprosthesis is recommended if the patient’s life expectancy is lower than the presumed durability of the bioprosthesis [32].

The potential for surgical or transcatheter reintervention is another important aspect. A mechanical prosthesis should be considered in patients for whom future redo valve surgery or TAVR would be a high risk. Assessing the thrombotic and bleeding risks of the patient is mandatory. A bioprosthesis is recommended if good-quality anticoagulation is unlikely or if anticoagulant treatment is contraindicated due to high hemorrhagic risk, such as that determined by comorbidities or previous major bleeding [32].

In general, biological valves are preferred over mechanical valves in hemophiliacs because long-term anticoagulant therapy is avoided. A preference for biological valves is also advocated by the latest clinical practice guidance document on antithrombotic treatment in patients with hemophilia [41].

In line with the newest recommendation, in hemophiliacs ineligible to receive a bioprosthetic valve because of age or a high risk of reintervention, the use of the mechanical On-X valve might be considered [41]. Due to its unique material and design, this valve is less susceptible to thrombosis than other mechanical heart valves. Based on PROACT (Prospective Randomized On-X Anticoagulation Clinical Trial) results, the On-X mechanical valve can be safely used with a lower intensity of anticoagulation with warfarin (INR 1.5–2 vs. standard INR 2–3), starting three months after aortic valve replacement [42,43]. It must be emphasized that the PROACT Mitral randomized trial failed to demonstrate the non-inferiority of the composite primary endpoint (thromboembolism, valve thrombosis, or bleeding) of low-dose warfarin (INR 2.0–2.5) vs. standard-dose warfarin (INR 2.5–3.5) in patients with an On-X prosthesis, who were >3 months post-mitral valve replacement [44].

Data available show that hemophiliacs younger than 65 years received mechanical mitral prostheses as recommended for the general population. Most hemophiliacs aged 50–65 years received biological aortic prostheses, paralleling the trend of the general population. There were a few exceptions. A 47-year-old patient with severe HA [18] and a 23-year-old patient with severe HA and inhibitors [12] received aortic bioprostheses. A Bentall operation with a biologic composite graft was performed on a 43-year-old patient with severe HA [22]. These approaches reflect the struggle to avoid long-term anticoagulation in patients with severe hemophilia.

## 4. Surgical Procedures

The majority of patients underwent classic surgeries, which included full sternotomy, CPB, cardioplegia, and direct approach of the heart. The published literature constantly emphasizes that the success of these surgeries results from meticulous preoperative planning and close collaboration within the multidisciplinary team: cardiac surgeon, anesthesiologist, hematologist, transfusion medicine, perfusionist, laboratory, and nursing in intensive care units and wards [11]. Without a doubt, the most dynamic and challenging aspect of the surgery is the bleeding control. Regarding the operative protocol, the less invasive it is, the more it participates in achieving this goal.

A minimally invasive approach was adopted by Chamos et al. [14]. The surgical team used a 6 cm right anterior mini-thoracotomy through the second intercostal space to gain access to the proximal ascending aorta and the aortic valve. The valvular replacement was performed under peripheral CPB obtained through cannulation of the right femoral vessels. The recovery was uneventful. Odonkor et al. reported the successful performance of a minimally invasive mitral valve repair surgery under CPB in a hemophiliac with symptomatic MR [8]. Because the minimally invasive approach is associated with less postoperative bleeding and a reduced need for blood transfusions, this technique has significant value in patients with bleeding disorders [45].

The feasibility and safety of robotic MV repair in the general population have already been confirmed [46], including when performing other procedures simultaneously, such as atrial ablation or tricuspid valve repair [47,48,49]. Its lower invasiveness leads to a lower requirement for blood products, less postoperative discomfort and pain, and shorter length of hospital stay. Robotic surgery of the mitral valve emerged as a feasible surgical option for hemophiliacs as well. Tran et al. successfully used the Da Vinci telemanipulation system for mitral valve repair [17]. For robotic instrumentation, only five incisions from 8 to 20 mm in size in the right chest were necessary. The left femoral vessels were cannulated to perform peripheral CPB. The cardiac procedure was complex and included the placement of a neochord and a mitral annulus ring, suture ligation of the left atrial appendage, and pulmonary vein isolation using cryoablation. Except for a right pleural effusion, which required repeated drainage, there were no other complications. It should be emphasized that the procedure was performed on a patient with moderate hemophilia A (HA) and high-titer inhibitors, who also refused the administration of blood products. A young patient with mild hemophilia B (HB) was the second hemophiliac undergoing robotic valve surgery. Miller et al. corrected a mitral regurgitation by using five neochordae and inserting a mitral annulus ring [16]. The patient had an uneventful recovery.

Transcatheter aortic valve replacement (TAVR) emerged 20 years ago as a viable therapeutic option for patients with severe symptomatic aortic stenosis (AS) deemed unsuitable for surgical aortic valve replacement due to high or prohibitive surgical risk. Improvements in valve design, materials, and delivery systems along with increasing experience in performing these procedures led to the expansion of the spectrum of indications. Several categories of patients with severe AS are now eligible for TAVR, as well as patients with aortic insufficiency and bioprosthesis degeneration [32]. A wide range of transcatheter heart valves was developed for the treatment of calcific AS—balloon-expandable, self-expandable, and mechanically expandable valves—allowing an individualized therapeutic approach [50]. Newly developed valves with active fixation mechanisms that can anchor onto non-calcified native valves are used to treat patients with aortic regurgitation. The first reported TAVR procedure in a patient with hemophilia is relatively recent. An 84-year-old patient with mild HA and a high burden of comorbidities—hypertension, type 2 diabetes, transient cerebral ischemic event, stented stenosis of the left internal carotid artery, quadruple CABG—successfully underwent transfemoral TAVR for severe AS complicated with recurrent exacerbations of heart failure (HF) [20]. Since TAVR is associated with a lower risk of bleeding complications compared to surgical aortic valve replacement [51], it is certainly a treatment method to consider in hemophiliacs.

In several patients, aortic valve replacement and CABG were performed concurrently. As recent data show, for patients who require both CABG and aortic valve replacement, the future lies in off-pump CABG with concomitant TAVR. This approach is a less invasive feasible option for patients presenting with a high-risk status [52].

## 5. Overview of the Surgical Protocol

Standard CPB, established by cannulation of the ascending aorta and the right atrium, is frequently used [30,39]. Other approaches, adapted to the type of surgical intervention performed, parallel those used in the general population. Femoral arterial and venous cannulation were chosen in a patient undergoing minimally invasive aortic valve replacement [14] and in two patients undergoing robotic mitral valve repair [16,17]. CPB was established between the right atrium and the innominate artery in a patient with acute dissection of the ascending aorta treated by a Bentall operation [25]. Cannulation of the right atrium and femoral artery was chosen for a patient with Marfan syndrome undergoing a Bentall operation for aortic regurgitation, dilated aortic root, and ascending aorta [22]. Internal jugular vein catheterization [5,8,21] and radial artery catheterization were also used [21]. However, most published cases only state that CPB/extracorporeal circulation was established without making any specific comments.

Anticoagulant treatment during CPB is mandatory and it was given in all cases. Unfractionated heparin (UFH) was used, guided by activated clotting time (ACT). ACT is the gold standard for measurements of heparin anticoagulation during CPB. In patients in the general population, ACT > 300 s is considered safe for cannulation and ACT > 400 s is recommended for going on bypass [53]. ACT > 480 s is required for going on deep hypothermic circulatory arrest, needed for complex procedures involving the aortic arch. Most institutions have ACT targets between 400 and 500 s, although lower or higher targets have been used [54]. In hemophiliacs, most ACT targets fall within this range. Lower targets, such as ACT ≥ 300 s [5] or >250 s [7], are rarely reported. At the end of CPB, protamine sulfate is administered to neutralize UFH and to bring ACT to normal [39].

Coagulation factor replacement aiming for complete correction is recommended before surgery [55]. The amount of coagulation factor used varies widely, depending on the severity of hemophilia, type of the coagulation factor, method of administration, presence of hemorrhagic complications, and experience of the center where the procedure was performed. In general, perioperative replacement therapy is performed with either intermittent boluses or continuous infusion. Continuous infusion seems to have additional benefits over intermittent boluses. A more stable plasma factor level with fewer peaks and troughs may lower the incidence of bleeding complications, and hence the use of blood products. Moreover, a possible reduction in the total coagulation factor dose could be achieved [8]. In all cases, the replacement of the coagulation factor was extended into the postoperative period, with the duration of FVIII administration varying from 1 to 24 days, depending on the patient’s characteristics and the presence or lack of bleeding complications [14]. Mannucci et al. recommended full clotting factor correction for 10 days after valvular surgery [31].

Management of blood products is also challenging. Some surgical teams reported the use of a cell saver and autologous blood transfusion [5,7,8,16,24,28,35,39]. As a general approach, replacement of coagulation-related variables after CPB with banked blood products should be directed at correcting specific deficits. Red blood cells, platelets, fresh frozen plasma, and fibrinogen concentrate are used depending on the need. Fresh frozen plasma is ineffective for FVIII replacement but helps to correct generalized factor deficiencies [8,22]. Platelet transfusion should be considered when the platelet count is <80–100 × 10^3^/µL after excluding FVIII and other factor deficiencies [8]. In bleeding associated with fibrinogen levels <200 mg/dL, administration of cryoprecipitate or fibrinogen concentrate may be preferable to plasma to avoid volume overload [8].

In the general population, high thrombotic complication rates were associated with the use of recombinant activated coagulation factor VII (rFVIIa) and factor VIII inhibitor bypassing activity (FEIBA) in the management of refractory post-CPB bleeding [56]. Although rFVII and FEIBA are used in patients with HA and inhibitors [8], caution is advised in the use of either product or prothrombin complex concentrates (PCCs) in HA patients experiencing excessive hemorrhage secondary to post-CPB coagulopathy. Because the levels of endogenous anticoagulants may be severely decreased, thus affecting the plasma half-lives of activated procoagulant enzymes, there is a risk of an unwanted thrombotic event [8,57]. One article reported the use of rFVII and another the use of PCC. A patient with severe HA and aortic dissection was initially misdiagnosed as having acute coronary syndrome and was given antiplatelet drugs—aspirin, clopidogrel, and tirofiban [25]. After the surgical intervention, blood loss occurred even though the FVIII level was normal. He required the administration of large amounts of blood products and rFVII to control the bleeding. The second case was an HB patient undergoing a Bentall operation under eftrenonacog alfa [24].

Patients with hemophilia have impaired clot generation and stability because the fibrin they produce is more sensitive to fibrinolysis, and inhibition of thrombolysis by thrombin is diminished. Therefore, epsilon aminocaproic acid (EACA) and tranexamic acid, both with antifibrinolytic effects, are used as hemostatic adjuvants in cardiac surgical hemophilic patients to decrease postoperative blood loss and blood transfusion requirement [8]. EACA and tranexamic acid are both lysine analogs. They interfere with the binding of plasminogen to fibrin, which is a necessary step in plasmin generation. Thereby, EACA and tranexamic acid inhibit fibrin degradation [58]. Tranexamic acid acts synergistically with factor VIII in patients with HA, thus increasing clot resistance to fibrinolysis [25]. EACA and tranexamic acid are both used perioperatively in heart valve replacement surgery in hemophiliacs. Tang et al. generally advocate for the use of tranexamic acid in all hemophilia patients requiring major surgery [30].

## 6. Risk of Developing Inhibitors

Patients with hemophilia require the administration of the deficient clotting factor either to prevent bleeding (prophylaxis) or to stop it (on-demand treatment). The development of anti-FVIII or FIX antibodies is a known complication of this treatment. Up to 30% of naïve patients with severe HA and about 9% of those with mild and moderate HA will develop inhibitors (alloantibodies) [59]. The development of inhibitors in HB patients is much rarer. Only 1.5–3% of all HB patients will develop inhibitors [60]. Inhibitors usually occur during the first 20–30 days from the initiation of treatment.

While the risk of developing inhibitors is highest during childhood in patients with severe hemophilia, in those with mild and moderate disease, inhibitors arise during adulthood and in the elderly, especially in those receiving large amounts of clotting factor concentrates for surgery, and particularly in those who have not been previously exposed to factor products [31]. In approximately 30–40% of patients, the inhibitors persist. This has long-term consequences because inhibitors make the management of any bleeding and surgical intervention very difficult.

Cardiac surgery requires normalization of the hemostasis before the procedure. Evidence shows that optimizing factor levels pre-, intra-, and postoperatively offers outcomes similar to those of patients without bleeding disorders [61]. Although there are no specific recommendations for cardiac surgery, the World Federation of Hemophilia recommends achieving a peak FVIII level of 80–100% preoperatively, then 60–80% for the first 1–3 days following major surgery, then 40–60% for days 4–6, then 30–50% for days 7–14 [62]. In patients with HB, a peak FIX level of 60–80% is recommended preoperatively, then 40–60% for the first 1–3 days following major surgery, then 30–50% for days 4–6, then 20–40% for days 7–14 [62].

Despite substantial efforts made, the mechanisms of inhibitor onset are not fully elucidated yet. However, the particularities of coagulation factor replacement treatment have a significant impact. In patients with HA, the source and type of FVIII products and treatment intensity are among the major contributors [63]. Recombinant FVIII products appear to have a higher immunogenicity than the plasma-derived products, and of the FVIII recombinant products, the second-generation, full-length products seem to be the most immunogenic in previously untreated patients [64,65,66]. In patients with HB, intermediate- and high-purity plasma-derived FIX concentrates were associated with increased inhibitor incidence compared to recombinant products [60].

The World Federation of Hemophilia recommends inhibitor testing before surgery, in cases of suboptimal postoperative responses to clotting factor concentrate replacement therapy and in any patient who is intensively treated (i.e., for more than 5 consecutive days) and within 4 weeks of the last infusion [62].

As far as the available literature shows, few patients with hemophilia and inhibitors underwent cardiac surgery for valvular disease [12,17]. One patient with HA had high-titer inhibitors of 12.4 BU before surgery, which declined to 6.2 BU at a 6-month follow-up [17]. The second patient had low-titer inhibitors, and due to the absence of bleeding during follow-up, the reevaluation of the titer was not considered necessary [12]. Therefore, careful planning of replacement therapy led to a favorable outcome. Furthermore, although large amounts of clotting factor were required perioperatively, no patient was reported to have developed inhibitors or symptoms suggestive of inhibitors. The case published by Ghosh et al. [4] is unique in that the patient undergoing cardiac surgery was undiagnosed with hemophilia. The disease was confirmed after surgery, in the context of severe bleeding. The patient developed low-titer FVIII inhibitors during the postoperative period, as he received large amounts of different blood products in a sustained attempt to control major and repeated bleeding.

## 7. Antithrombotic Treatment

Mechanical prostheses require life-long anticoagulant treatment with VKA [32]. The INR guides the treatment, with a target that depends on the thrombogenicity of the prosthesis and patient-related risk factors. Of note, a higher INR is required for mitral prostheses than for aortic prostheses. Among hemophiliacs, all those treated by implantation of a mechanical prosthesis received anticoagulant treatment with VKA. A reduced intensity of anticoagulation was used only in two patients, with a target INR of 1.5–2 [4,13]. The outcome was favorable over a short follow-up period.

Biological prostheses are less thrombogenic, allowing a short duration of antithrombotic treatment. ESC Guideline recommends VKA for 3 months for a mitral bioprosthesis. After surgical implantation of an aortic bioprosthesis, either aspirin or VKA should be considered for 3 months [32]. In hemophiliacs, the therapeutic approach varied widely. While some authors did not give antithrombotic therapy after discharge, others recommended low-molecular-weight heparin (LMWH) for a duration ranging between 1 and 3 months. Few authors recommended aspirin. One patient on aspirin [39] and one on LMWH [30] experienced gastrointestinal bleeding and were rehospitalized.

Transcatheter aortic bioprostheses require only antiplatelet treatment. A meta-analysis showed a significant increase in major or life-threatening bleeding with dual-antiplatelet therapy over aspirin at 30 days, with no difference in ischemic outcomes [67]. Recently published results of the POPular TAVI trial are in line with previous findings. Bleeding and the composite endpoint of bleeding or thromboembolic events were reduced with aspirin compared to dual-antiplatelet therapy [68]. As a consequence, life-long single-antiplatelet therapy is currently recommended after TAVR [32]. The hemophiliac treated with TAVR received monotherapy with aspirin [20].

VKA is recommended for the first 3 months after mitral valve repair due to an increased risk of thrombosis on non-endothelialized repair components and a high incidence of atrial fibrillation [32]. However, observational data suggest that the risk of thromboembolism after mitral valve repair is comparable between aspirin and VKA [69]. In the available literature, a clear preference for using aspirin in hemophiliacs was noted.

Antithrombotic treatment is feasible in hemophiliacs only if the coagulation factor level is consistently maintained above a specific trough limit. Of note, antithrombotic therapy is not recommended in hemophiliacs with inhibitors not using emicizumab. A minimum trough FVIII/FIX level of 1–5% is required for single-antiplatelet therapy with aspirin or clopidogrel [41]. Therefore, patients with severe hemophilia must receive regular clotting factor prophylaxis, aiming to maintain factor levels ≥ 1%. A minimum trough FVIII/FIX level of 20% is necessary for dual-antiplatelet therapy and oral anticoagulation with VKA, with INR levels 2–3. The most recent guidance document states that hemophiliacs with baseline clotting factor levels ≥ 20% can safely start oral anticoagulation and additional clotting factor prophylaxis is not recommended [41]. If factor levels are < 20%, the recommendations differ depending on the estimated duration of the treatment. If short-term anticoagulant treatment is needed, clotting factor prophylaxis will be adapted to maintain factor levels ≥ 20%. If long-term anticoagulant treatment is considered, it is recommended not to start with oral anticoagulation and consider that hemophiliacs are naturally anticoagulated. This concept is supported by studies of thrombin generation, which allowed a deeper understanding of hemostasis in hemophiliacs. Hemophiliacs with factor levels < 10% appeared anticoagulated similar to patients on VKA at therapeutic INR. Those with factor levels of 10–20% appeared anticoagulated, similar to patients on VKA at a lower INR of 1.5–1.9. However, for factor levels > 20%, the interindividual variance was wide and overlap with normal coagulation profile was observed [41]. This might explain why some patients—mostly with severe hemophilia or inhibitors—did not receive antithrombotic treatment after discharge.

## 8. Final Considerations

Due to genetic determinism of coagulation FVIII/FIX deficiency, hemophiliacs maintain an increased risk of bleeding throughout their lives. While hemophiliacs with mild (factor activity levels 5–40%) and moderate disease (factor activity levels 1–5%) usually bleed in the context of trauma or surgery, patients with severe hemophilia (factor activity levels <1%) may bleed spontaneously [62].

During surgery, congenital coagulopathy is exacerbated due to cardiopulmonary bypass, intravenous heparin administration, hypothermia, and acute normovolemic hemodilution. There is also the risk of bleeding at the level of sternotomy and anastomoses. Coagulation factor correction is always necessary, but the perioperative requirement is difficult to standardize for multiple reasons. Some are patient-dependent, such as the type and severity of hemophilia, the presence of ongoing coagulation factor prophylaxis, the existence of inhibitors, and coagulation factor clearance. Others relate to the coagulation factor product used (varied half-lives), the surgical intervention (use of cardiopulmonary bypass, intraoperative bleeding, hemodilution due to infusion of large amounts of colloids and/or crystalloids, need for transfusion, target factor level), and the type of antithrombotic therapy required postoperatively (antiplatelet or anticoagulant).

Individualization of treatment within a multidisciplinary team is the cornerstone that ensures the success of any procedure [39]. With careful planning, cardiac surgical interventions can be carried out safely in these high-bleeding-risk patients [1]. Hemophiliacs experience quite similar clinical outcomes when compared with the general population [30]. However, a preference for less invasive procedures of equal efficacy emerges. Valvular repair and bioprosthetic valves should be preferred over mechanical valves to avoid life-long anticoagulation. In patients ineligible to receive a bioprosthetic valve, the use of the mechanical On-X valve might be considered because it requires less intense anticoagulation beyond 3 months of treatment [41]. Given the trend towards the implementation of general population guideline recommendations in hemophiliacs, the benefits of biologic prostheses and their progressively improved durability characteristics, and the technical progress of transcatheter interventions, it is expected that in the future, more hemophiliacs will be considered suitable to receive a biologic prosthesis.

In the long term, these patients must remain under double supervision of the cardiologist to monitor the valve prosthesis and of the hematologist to modulate the hemophilia therapy. When the situation requires it, the two specialists will work closely together for the antithrombotic therapy to be effective and safe.

Our review has several limitations. Firstly, the data come from case reports and short series of cases. It is most likely that the experience in real life is much greater, but it has not been published. Secondly, the main focus of the published articles was the perioperative management of hemostasis, with little emphasis on the type of valvular disease, the surgical technique, or the type of prosthesis implanted. Thus, we had difficulties in extracting the necessary data. However, our review is valuable because, for the first time, the available data on the modern surgical treatment of valvular disease in hemophiliacs have been synthesized and systematized.
